# BERT-based language model for accurate drug adverse event extraction from social media: implementation, evaluation, and contributions to pharmacovigilance practices

**DOI:** 10.3389/fpubh.2024.1392180

**Published:** 2024-04-23

**Authors:** Fan Dong, Wenjing Guo, Jie Liu, Tucker A. Patterson, Huixiao Hong

**Affiliations:** National Center for Toxicological Research, US Food and Drug Administration, Jefferson, AR, United States

**Keywords:** pharmacovigilance, social media, drug, adverse event, language model (LM)

## Abstract

**Introduction:**

Social media platforms serve as a valuable resource for users to share health-related information, aiding in the monitoring of adverse events linked to medications and treatments in drug safety surveillance. However, extracting drug-related adverse events accurately and efficiently from social media poses challenges in both natural language processing research and the pharmacovigilance domain.

**Method:**

Recognizing the lack of detailed implementation and evaluation of Bidirectional Encoder Representations from Transformers (BERT)-based models for drug adverse event extraction on social media, we developed a BERT-based language model tailored to identifying drug adverse events in this context. Our model utilized publicly available labeled adverse event data from the ADE-Corpus-V2. Constructing the BERT-based model involved optimizing key hyperparameters, such as the number of training epochs, batch size, and learning rate. Through ten hold-out evaluations on ADE-Corpus-V2 data and external social media datasets, our model consistently demonstrated high accuracy in drug adverse event detection.

**Result:**

The hold-out evaluations resulted in average F1 scores of 0.8575, 0.9049, and 0.9813 for detecting words of adverse events, words in adverse events, and words not in adverse events, respectively. External validation using human-labeled adverse event tweets data from SMM4H further substantiated the effectiveness of our model, yielding F1 scores 0.8127, 0.8068, and 0.9790 for detecting words of adverse events, words in adverse events, and words not in adverse events, respectively.

**Discussion:**

This study not only showcases the effectiveness of BERT-based language models in accurately identifying drug-related adverse events in the dynamic landscape of social media data, but also addresses the need for the implementation of a comprehensive study design and evaluation. By doing so, we contribute to the advancement of pharmacovigilance practices and methodologies in the context of emerging information sources like social media.

## 1 Introduction

Within the intricate process of drug development, two critical elements emerge: efficacy and safety. Efficacy, denoting as a drug’s capacity to achieve the intended therapeutic effect, is undeniably crucial. However, safety holds an equal, if not more significance in the realm of drug development. Ensuring that a drug not only performs its intended function, but also upholds its safety profile is fundamental to the drug development process. The emphasis on safety extends beyond the initial stages of production; it perseveres even after the drug has been introduced to the market. Regulatory approval and subsequent administration to patients do not absolve the responsibility. The continuous monitoring of the medication’s safety remains an essential obligation, constituting a vital aspect of pharmacovigilance or drug safety surveillance. Monitoring drug safety poses a formidable challenge. Given the enormity of this task, there is a growing trend towards utilizing automated systems to adeptly surveil potential drug safety issues throughout the drug’s development cycle (1).

Drug safety surveillance is the process of monitoring, evaluating, and improving the safety of medications, playing a crucial role in ensuring patient well-being by identifying, assessing, understanding, and preventing adverse effects or other drug-related issues. In previous literature, the FDA’s Adverse Event Reporting System (FAERS) stands out as a crucial tool for drug safety surveillance (2, 3). This database aggregates information about adverse events and medication errors reported to the FDA, drawing from healthcare professionals, consumers, and manufacturers. FAERS is designed to support FDA’s post-market safety surveillance program for drug and therapeutic biologic products. The Algarni study serves as an exemplary case of utilizing FAERS data for drug safety surveillance, specifically examining adverse events associated with direct-acting antivirals base on post-market data (4).

While FEARS provides high-quality structured data crucial for establishing the safety and efficacy of new medications in a controlled and rigorous manner, its limitations include a restricted number of participants with limited demographic diversity, and results are only available after the trials completion. Consequently, relying solely on FEARS data for monitoring drug safety proves less effective. Instead, social media sites have become important in complementing traditional drug safety surveillance (5, 6).

During the COVID-19 pandemic, numerous pharmacological agents were suggested as potential treatments, yet their efficacy remains an area of ongoing investigation. Social media channels empower patients to share their personal experiences with these drugs. The immediacy and diversity of these patients, representing a globally distributed population, offer a rich, real-time data source that significantly enhances the breadth and depth of drug safety surveillance (7, 8). For example, Guo et al. focused on identifying occurrences of COVID-19 and related symptoms as conveyed by users on Reddit (9). Simultaneously, Yu and Vydiswaran utilized natural language processing (NLP) algorithms to identify and classify Twitter posts related to drug adverse events (10). This exemplifies the application of advanced computational methods in drug safety surveillance. In summary, while FAERS adopts a conventional and structured approach to drug safety surveillance, social media offers an extensive and unstructured dataset capable of delivering real-time insight. Each approach possesses its strengths and weaknesses, and ideally, they should be used synergistically in pharmacovigilance to provide a more comprehensive understanding of drug safety (11).

Social media has emerged as a valuable resource in the realm of drug safety surveillance, providing a platform for real-time, patient-generated data on drug use and its associated adverse events. However, the integration of social media data into drug safety surveillance encounters various challenges and limitations. The primary challenge lies in data quality. Social media posts are unstructured and exhibit considerable variability in terms of quality and accuracy. Assessing the reliability of these reports is challenging, given that they often utilize informal language and lack clinical validation.

Analysis of social media data poses another challenge due to the substantial amount of irrelevant information (noise) present, necessitating sophisticated filtering to extract useful data. Unlike the structured FAERS database, social media lacks standardization in the reporting of adverse events, leading to inconsistencies and difficulties in data interpretation. Establishing a direct causal relationship between a drug and an adverse event base on social media reports is complex, as these platforms often lack detailed patient history and medication usage information.

Looking towards future regulatory and integration efforts, incorporating social media data into established pharmacovigilance systems poses regulatory challenges and issues in combining data from these non-traditional sources with existing databases. In conclusion, while social media offers a novel and potentially valuable source of information for drug safety surveillance, its effective use in pharmacovigilance requires overcoming substantial challenges, particularly in the areas of data quality, analysis, and regulatory considerations (12, 13). Continued research and development in this domain are imperative for addressing these limitations.

The emergence of social media has revealed new avenues for monitoring and extracting information about adverse events, a vital element of drug safety surveillance. A scoping review was conducted to evaluate the effectiveness of social media in detecting adverse events and integrating them into pharmacovigilance (14). This study highlights the lack of consensus on the role of social media in pharmacovigilance when compared to traditional data sources, noting that most research focuses more on the detection of adverse events than on their extraction.

Exploring adverse event extraction models from the literature, a machine learning approach was employed to trace COVID-19 vaccine adverse events using Twitter data (15). This research holds significance for its focus on extracting specific adverse events, thereby highlighting the potential of social media for real-time vaccine adverse event monitoring. Similarly, a deep learning pipeline was established to detect signals of adverse events related to dietary supplements from Twitter, focusing on specific adverse event types and comparing the findings with known adverse events from a dietary supplement knowledge base (16). However, both studies primarily concentrated on a limited range of specific adverse events.

Furthermore, an ALBERT-BiLSTM-CRF model was introduced for named entity recognition from adverse drug reaction information online, utilizing Chinese medical data (17). This study demonstrates the effectiveness of combining deep learning with NLP for adverse event entity recognition, although it lacks comprehensiveness in terms of tuning and evaluation. An NLP framework, the adverse drug reaction detection framework, was employed to identify adverse drug reactions in drug reviews on social media (18). This paper underscores the potential of social media as a valuable source for detecting unreported adverse drug reactions, particularly by leveraging existing NLP tools for analysis. However, the study does not provide detailed information on the implementation of the NLP tool MetaMap.

In conclusion, drug adverse event extraction models employ diverse approaches: some focus on specific adverse events, while others pursue more generalized extraction, encountering limitations such as language constraints (e.g., Chinese data) or reliance on existing NLP tools. Moreover, many studies lack thorough details in terms of implementation and evaluation.

Our model aims to address these limitations by presenting a comprehensive design for the extraction of adverse events from social media. It incorporates detailed implementation and evaluation plans, intending to meet the need for a robust and efficient system for drug adverse event extraction in pharmacovigilance. By bridging these gaps, our model holds the promise of significantly enhancing drug safety monitoring by harnessing the innovative potential of social media data.

## 2 Methods

### 2.1 Study design

**Figure 1:**
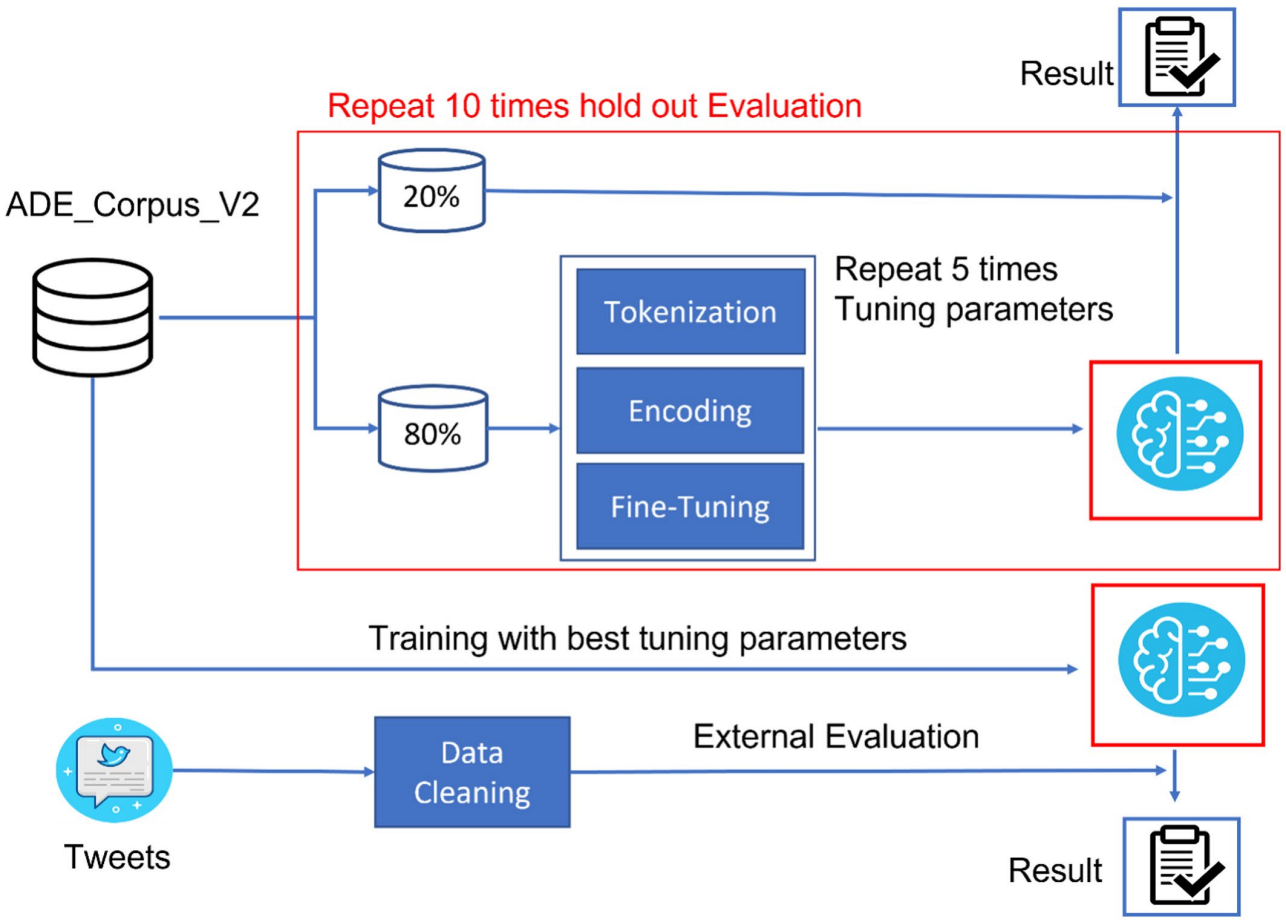
Study design for BERT-based adverse events extraction from social media data.

The study was structured to develop a robust bidirectional encoder representation from a transformers (BERT)-based language model capable of accurately extracting adverse events from social media data. As illustrated in Figure 1, the study design encompassed data processing, model training, and evaluation. ADE_Corpus_V2 data (19), containing annotated adverse event terms from the medical literature, and Tweets data with human-labeled adverse event terms from SMM4H (20), were collected and processed in this study. ADE_Corpus_V2 data were used to train the BERT-based adverse event extraction model.

In the model training procedure, 80% of the ADE_Corpus_V2 was allocated for parameter tuning, while the remaining 20% of the data were reserved for internal model evaluation. The detailed model training process involved three sequential steps: tokenization, encoding, and fine-tuning. This random split strategy was repeated 10 times for internal evaluation to ensure the stability and reliability of our model. Following five iterations to optimize fine-tuning hyperparameters, the complete ADE-Corpus-V2 dataset was used to train the final model with the fine-tuning hyperparameters. Subsequently, Tweets data were collected from SMM4H, underwent a cleaning process to remove noise and non-relevant content, and were used for external evaluation of our model.

### 2.2 Datasets

#### 2.2.1 ADE_Corpus_V2 dataset

The ADE-Corpus-V2 is a specialized dataset tailored for research in the field of pharmacovigilance, with a primary focus on identifying and extracting adverse drug events from text. This corpus serves as a valuable resource for developing and testing NLP models aimed at comprehending drug-related safety issues. Derived from a variety of sources, predominantly medical case reports and summaries, the corpus contains rich information regarding drug usage and associated adverse events. The dataset is meticulously annotated, highlighting instances of adverse drug events and the drugs associated with these events. These annotations play a crucial role in training NLP models to recognize and extract similar information from unstructured text.

**Figure 2:**
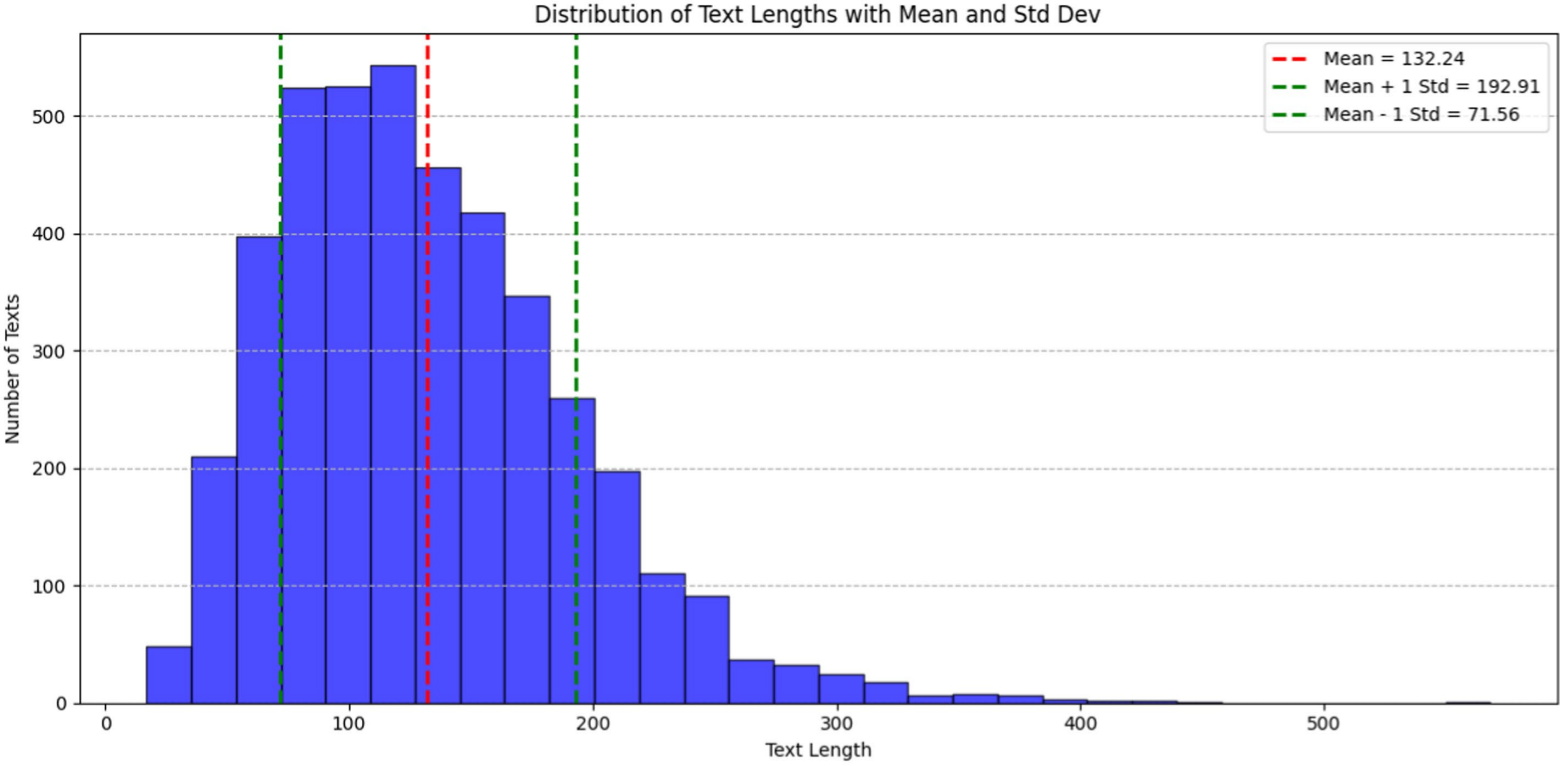
Distribution of text lengths for ADE-Corpus-V2 dataset.

In Figure 2, the histogram illustrates the distribution of text lengths within the ADE-Corpus-V2 dataset. The X-axis corresponds to document length, while the Y-axis denotes the number of documents. The red dashed line denotes mean text length, standing at 132.24 characters. Additionally, the green dashed lines represent one standard deviation (Std Dev) from the mean, with values at 71.56 characters (Mean − 1 Std Dev) and 192.91 characters (Mean + 1 Std Dev). These lines offer statistical insight into the variability and spread of text lengths in the dataset. The majority of texts fall within the range of 50 to 250 characters, underscoring the typical length of entries in the corpus.

The ADE-Corpus-V2 dataset undergoes systematic double annotation in multiple rounds to ensure consistent annotations. All annotations are limited to sentence level, ensuring that the annotated drugs and the related adverse events co-occur only within individual sentences.

In essence, the ADE-Corpus-V2 dataset comprises a comprehensive collection of 4,271 documents, encompassing 5,063 drugs and 6,821 instances of adverse events Among these adverse events, it identified 3,341 unique occurrences, highlighting the dataset’s breadth and diversity in capturing various medical terms and patient experiences.

#### 2.2.2 SMM4H dataset

The SMM4H (Social Media Mining for Health) annotated tweets dataset for adverse event extraction is a curated collection of tweets that have been annotated for adverse drug events. These tweets, sourced from Twitter, present authentic user-generated content where individuals share their experiences with medications. A team of trained medical experts systematically labels the data following the SMM4H-SocialDisNER guidelines. They identify and categorize mentions of adverse events and associated drugs. Each tweet is given an adverse drug event label if it contains an adverse event mention and is linked to standardized medical terminology databases, such as MedDRA (Medical Dictionary for Regulatory Activities), for consistent terminology.

This dataset is invaluable for researchers and developers in the medical informatics field, enabling the development and evaluation of NLP algorithms and machine learning models for automatically detecting adverse drug events in social media. This emerging area is crucial in pharmacovigilance and public health monitoring.

**Figure 3:**
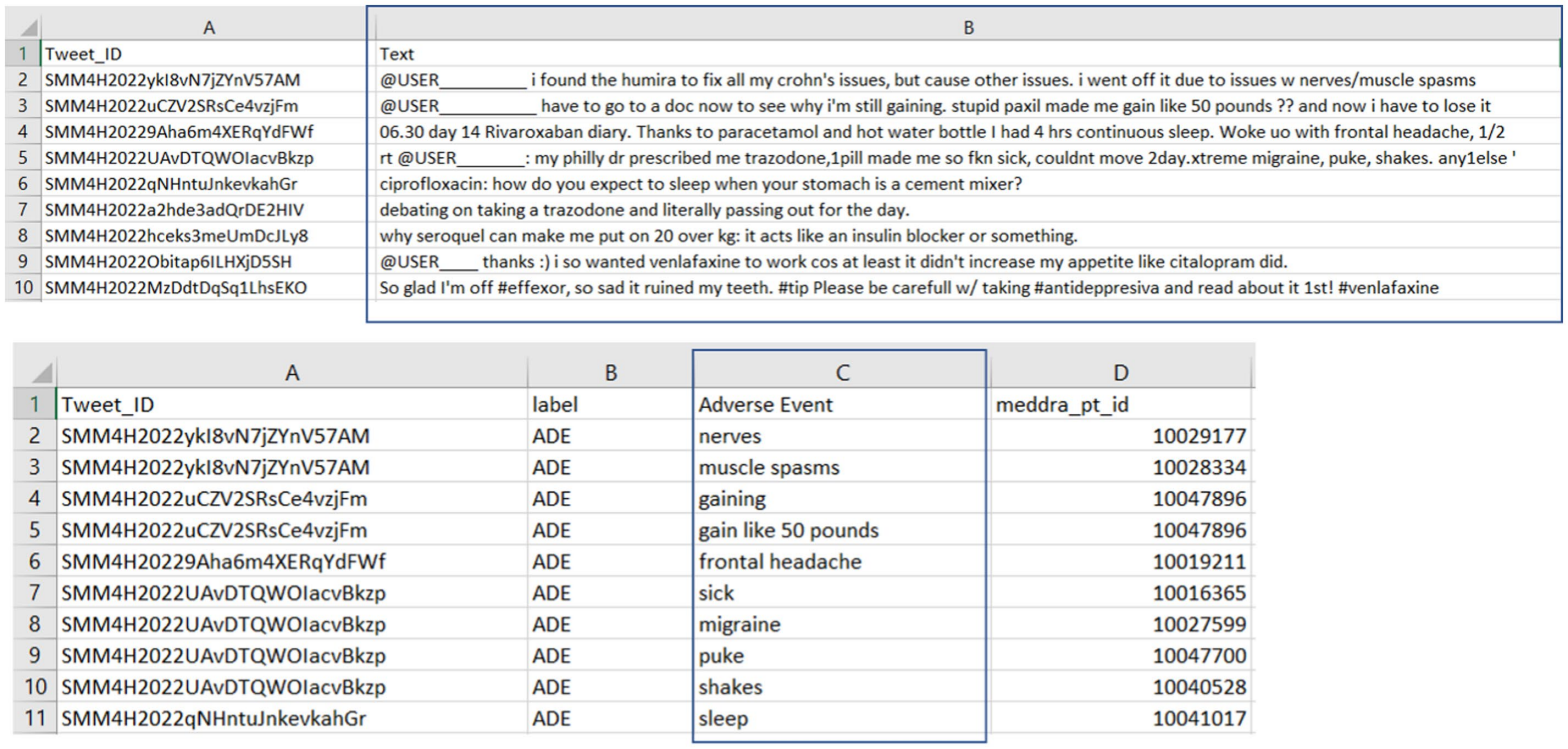
Overview of human-annotated adverse event tweets from the SMM4H public dataset.

The details of the SMM4H annotated tweets data are depicted in Figure 3. The upper section displays tweet IDs (Column A) and their respective tweet text content (Column B), while the lower section correlates these tweet IDs with the human-annotated adverse events (Column C). This structured representation underscores the meticulous process of curating and labeling real-world experiences of adverse events shared on social media, serving as a crucial resource for pharmacovigilance research and drug safety. In our study, tweets and labeled adverse events were extracted for external evaluation of our model’s adverse event extraction performance on tweets data. Our external evaluation contained 909 tweets, with 65 tweets bearing the adverse drug event label and 87 annotated adverse events within these tweets.

### 2.3 Data preprocess

We preprocessed the ADE-Corpus-V2 dataset and the SMM4H dataset using the following steps in our model development. For the ADE-Corpus-V2 dataset, we used random seeds values from the list (2, 12, 22, 32, 42, 52, 62, 72, 82, 92) and the train_test_split and random_state functions in the Python scikit-learn library. This allowed us to randomly split the dataset into 20% for testing and 80% for training, facilitating our internal evaluation.

For the SMM4H dataset, we removed irrelevant content from the origin tweets text data such as URLs, usernames (e.g., @mentions), non-texture elements like emojis and emoticons, and special characters outside the ASCII range (hexadecimal values from 00 to 7F). These exclusions were made because such content does not contribute to the adverse event detection. The resulting clean tweet text data were then used for external evaluation of our model.

This preprocessing is crucial for effective NLP and plays a pivotal role in ensuring the quality and accuracy of the information extracted by our model. The meticulous removal of irrelevant elements and the appropriate partitioning of the data are crucial steps that enhance the robustness and reliability of our model’s performance during both internal and external evaluations.

### 2.4 BERT-based-uncased details

The BERT-based-uncased model represents a specific variant of the BERT model, which has significantly transformed the landscape of NLP. Developed by Google researchers, BERT’s architecture enhances its ability to comprehend the contextual meaning of words within a sentence, surpassing the capabilities of its predecessors. This variant, the BERT-based-uncased model, proves exceptionally effective across a diverse range of NLP tasks, including sentiment analysis, named entity recognition, question answering, and language inference.

While BERT models undergo pre-training on extensive text corpora, their versatility lies in their capacity to be fine-tuned with additional layers for specific tasks. This adaptability makes them highly suitable for various NLP applications. The BERT-based-uncased model, in particular, comprises 12 transformer layers, also referred to as encoder layers, housing approximately 110 million parameters in total. These layers and parameters empower the BERT-based-uncased model to effectively process and understand the contextual nuances of language in various NLP tasks.

The considerable size of the model, as indicated by the number of layers and parameters, contributes to its robust and high-performance capabilities. However, it is essential to note that this attribute also renders the model computationally intensive. The power and effectiveness of the BERT-based-uncased model arise from its intricate architecture, enabling it to address intricate language understanding tasks, albeit with the computational demand inherent in its extensive design.

### 2.5 Model development

Developing a BERT-based model for extracting adverse events begins with text preparation through tokenization. BERT’s tokenizer dissects input sentences into smaller, identifiable pieces. Special markers, such as “CLS” for classification at the sequence’s start, “SEP” for separating sequences or sentences, and “PAD” for equalizing sequence lengths during batch processing, were introduced. These tokens were encoded into dense embeddings, endowing the model with the ability to discern the role, position, and contextual relationships of each word within the sentence.

In the fine-tuning phase, the model leverages a dense layer with SoftMax activation to meticulously classify each token, assigning labels such as B-AE (beginning of an adverse event) for the beginning of an adverse event entity, I-AE (inside of an adverse event) for subsequent parts, and O (outside of adverse events) for non-entity tokens. Table 1 shows the named entity recognition classes and their corresponding labels and meanings for our model. The CrossEntropyLoss function, a standard loss function for classification tasks, is employed during training to compare predicted probabilities with actual labels, crucial for calibrating the model’s weights.

**Table 1:** Named entity recognition classes and their corresponding labels.

Class	Label	Meaning
O	0	Token is not part of named entity (Adverse Event)
B-AE	1	Beginning of named entity (Adverse Event)
I-AE	2	Inside named entity (Adverse Event)

**Figure 4:**
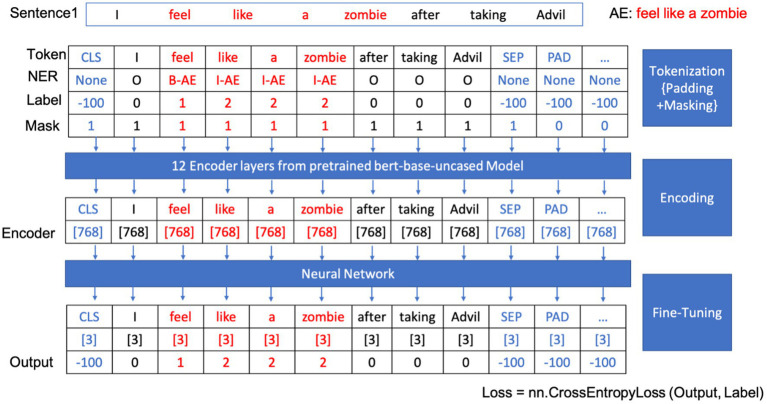
Workflow of the BERT-based adverse event extraction model.

Through iterative training and evaluation, the model becomes a precise tool for extracting adverse event data from new texts. Figure 4 outlines the workflow of our BERT-based adverse event extraction model with a simple example. Tokenization, padding, and masking were applied to the input sentences, and the resulting tokens were passed through 12 encoder layers of a pre-trained BERT-based-uncased model. The output, representing named entity recognition classes for each token, aids in identifying and extracting the adverse event mentions.

**Figure 5:**
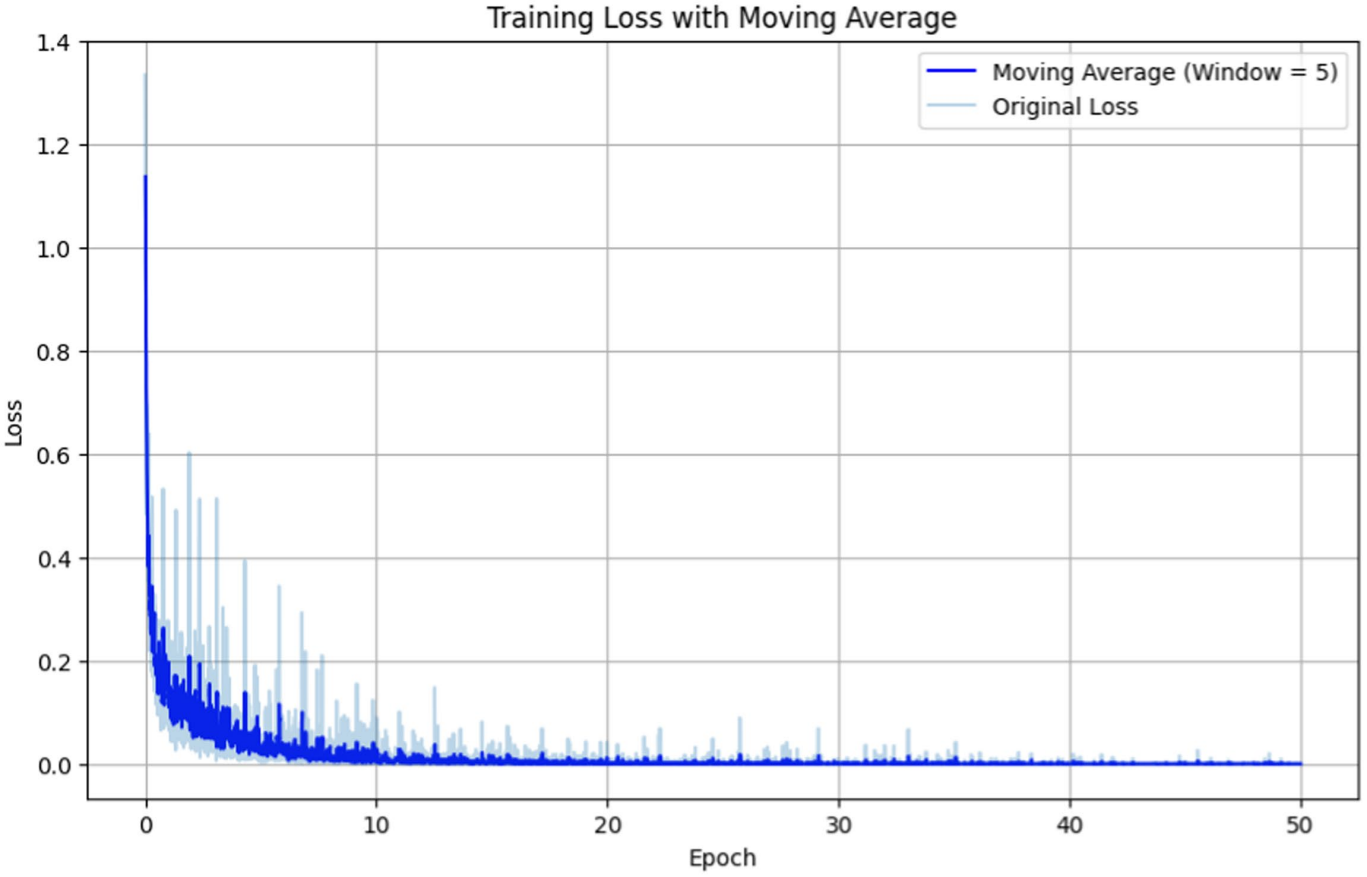
Training loss vs. epochs for fine-tuning the BERT-based adverse event extraction model hyperparameter epochs.

**Figure 6:**
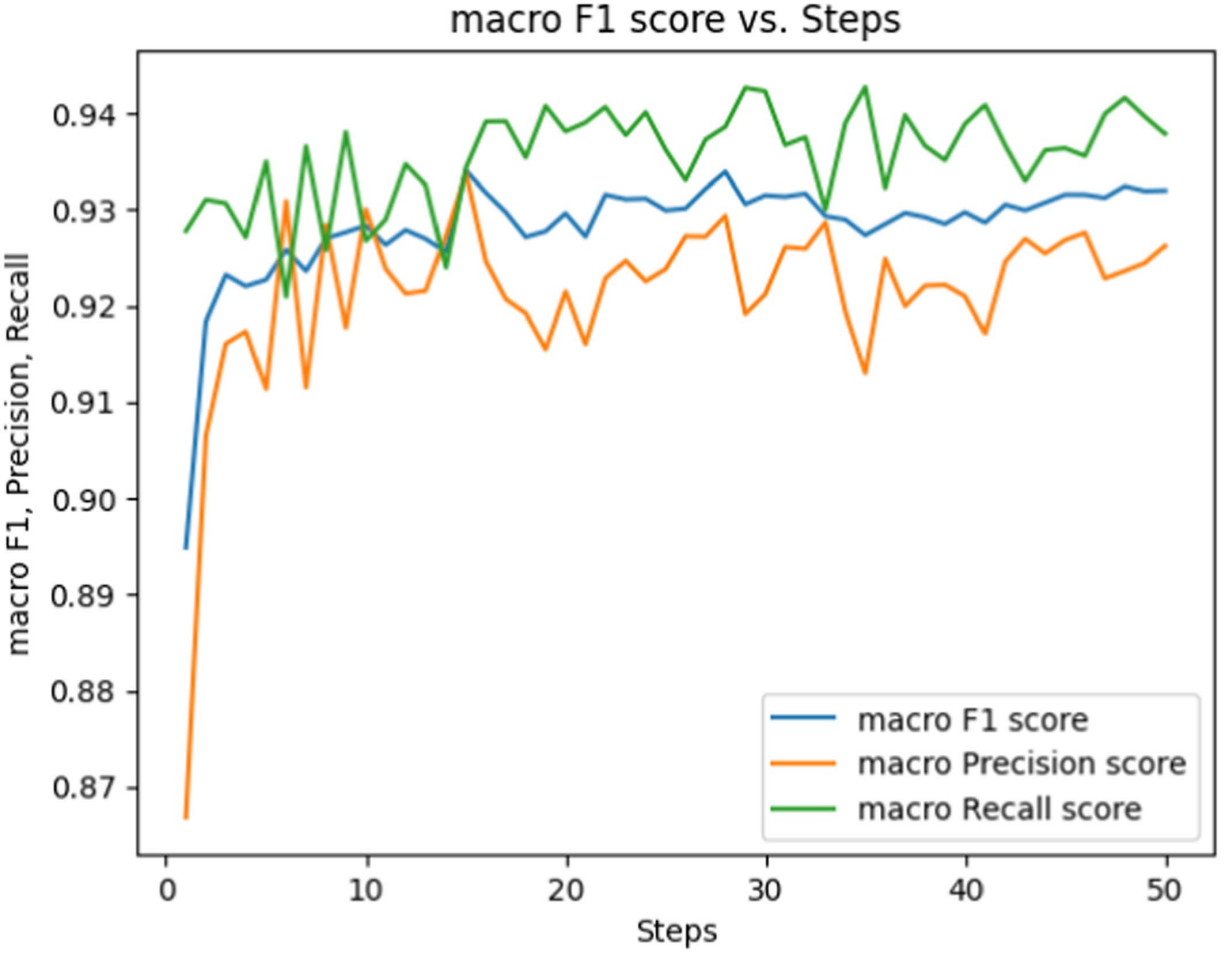
Macro F1, precision, Rrecall score vs. epochs for fine-tuning the BERT-based adverse event extraction model hyperparameter epochs.

Fine-tuning involves adjusting hyperparameters, with number of epochs, batch size, and learning rate being crucial. The term epochs refer to the number of times the learning algorithm iterates through the entire training dataset. Too few epochs may lead to underfitting, while an excessive number can lead to overfitting. After randomly partitioning the ADE-Corpus-V2 dataset into 80% for training and 20% for testing, we repeated the process five times to observe the training loss throughout epochs in Figure 5, and macro F1, precision, and recall scores over epochs in Figure 6, respectively.

In Figure 5 the original loss is depicted by the light blue line representing the immediate loss at each epoch. Simultaneously, the moving average (computed with a window size of 5) is illustrated by the dark blue line, providing a smoother representation of loss trends. The training loss exhibits a sharp decline in the initial epochs, followed by a plateau, indicating that the model is learning and subsequently stabilizing. The moving average of the loss gives a smoother curve, facilitating the discernment of overall trend beyond the noise of individual epochs.

Figure 6 showcases performance metrics over 50 epochs. The macro F1 score is depicted by the blue line, signifying the equilibrium between precision and recall. The macro precision score and macro recall score are represented by the orange and green lines, respectively. While macro F1, precision, and recall scores fluctuate during the training steps, they generally show an upward trend. This suggests an enhancement in the model’s capability to accurately label the data as it undergoes training.

Based on these observed trends, we opted for a duration of 50 epochs to strike a delicate balance between mitigating underfitting and avoiding overfitting. By the 50th epoch, the loss had reached a stabilized state, suggesting that further training would yield diminishing returns. Moreover, the performance metrics (F1, precision, recall) indicate that the model reached a performance plateau, implying that it had likely learned as much knowledge as possible from the training data. This specific number of epochs ensures the model is finely tuned to generalize to external data —a critical aspect for the successful development of an adverse events extraction model.

**Figure 7:**
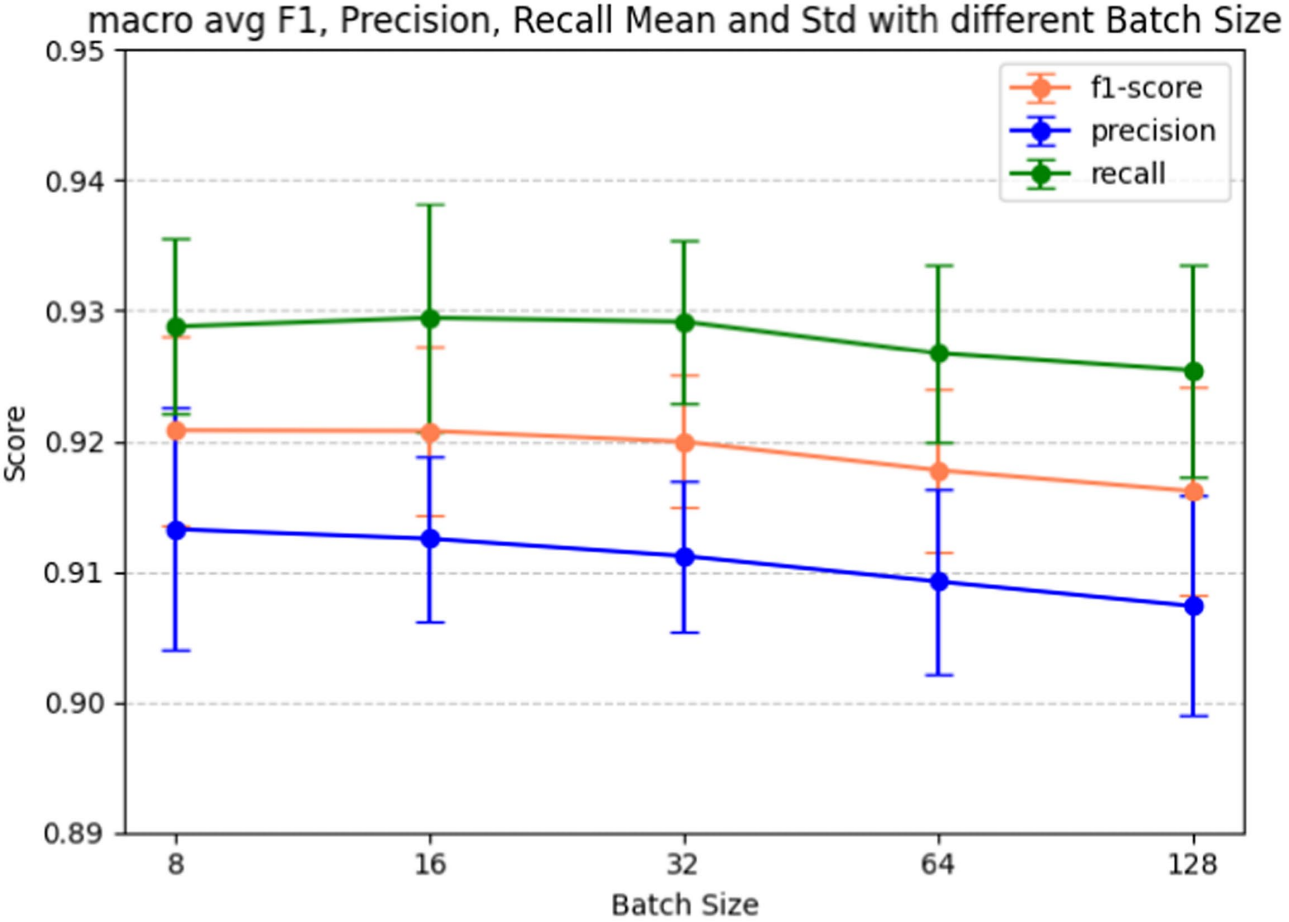
Macro F1, precision, recall score vs. batch size for fine-tuning the BERT-based adverse event extraction model hyperparameter batch size.

**Figure 8:**
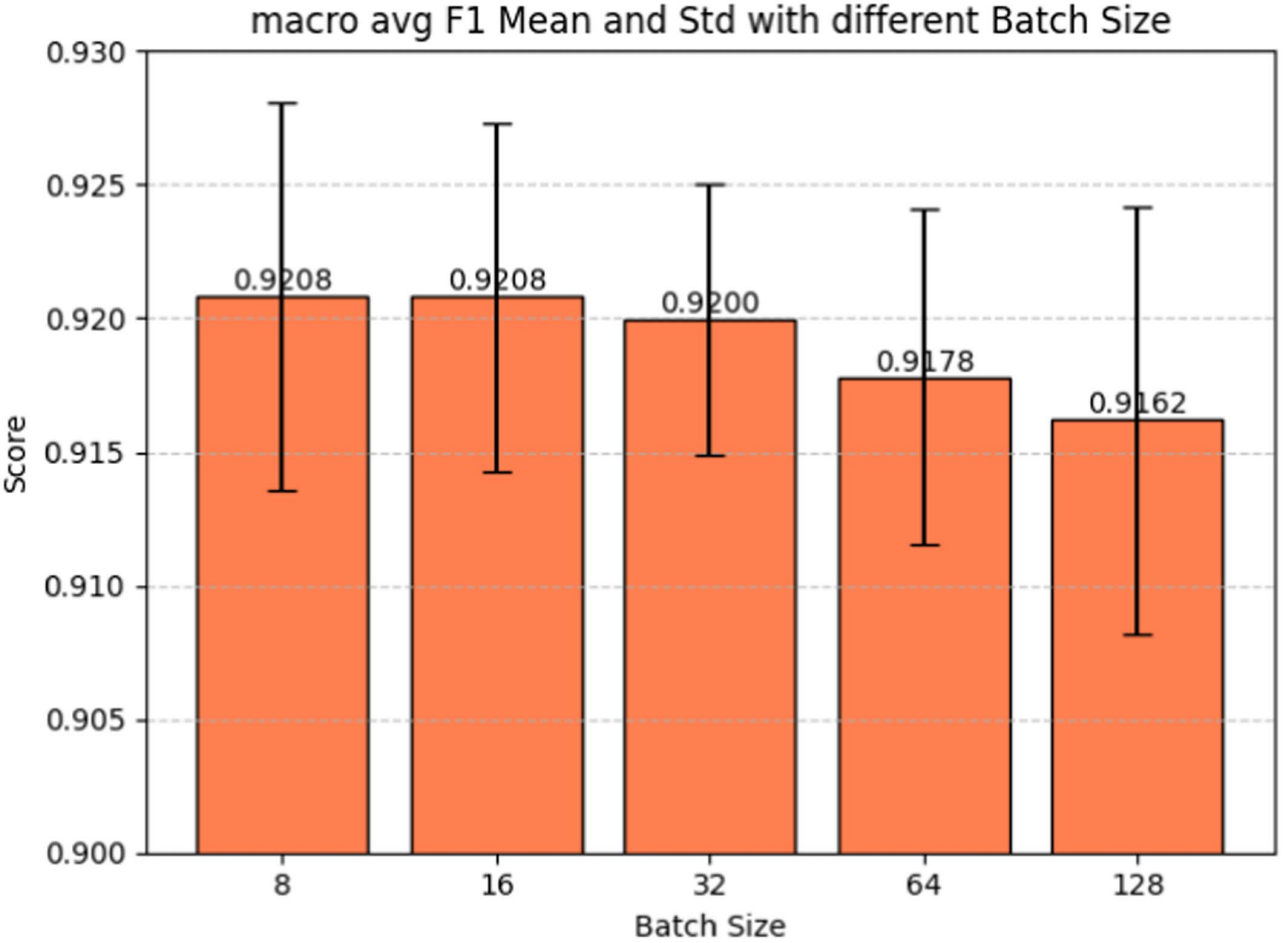
Macro average F1-score vs. batch sizes for fine-tuning the BERT-based adverse event extraction model hyperparameter batch size.

Batch size plays a pivotal role in determining how many examples the model encounters before updating its weights, influencing the variance of gradient estimation and overall memory consumption during training. Fine-tuning the batch size hyperparameter is also a crucial step in our model development, given its significant impact on the model’s learning dynamics and performance. Figure 7 shows the batch size tuning for the BERT-based adverse event extraction model. The chart portrays the macro-average F1-score (red), precision (blue), and recall (green) as functions of varying batch sizes (8, 16, 32, 64, 128). Error bars in the graph represent the standard deviation for each score at different batch sizes. Figure 8 shows the macro average F1-score across different batch sizes in the BERT-based adverse event extraction model. The bars represent the mean F1-scores for batch sizes of 8, 16, 32, 64, and 128, while the error bars depict the standard deviation.

A smaller batch size typically yields a more resilient gradient estimate, albeit with increased noise. This noise can be advantageous, imparting a regularizing effect and aiding the model in escaping local minima. On the contrary, larger batch sizes facilitate faster computation due to better hardware utilization, but they may lead to less generalizable models due to the potential for smoother optimization landscapes setting into suboptimal minima. A distinct stabilization of macro F1 scores, exhibiting minimal standard deviation and high accuracy, is evident at a batch size of 32. This observation designates it as the optimal batch size for achieving the best performance in the model.

Our final decision to select a batch size of 32 reflects a delicate balance between computational efficiency and the quality of gradient estimates. The choice suggests that this strikes the best trade-off between training speed and model performance, as substantiated by the F1 scores, precision, and recall values. It is plausible that the chosen batch size also ensures consistent training convergence and stability, considering that larger batch sizes might compromise performance or introduce increased variability, while smaller sizes may not yield substantial improvements to justify the additional computational cost.

**Figure 9:**
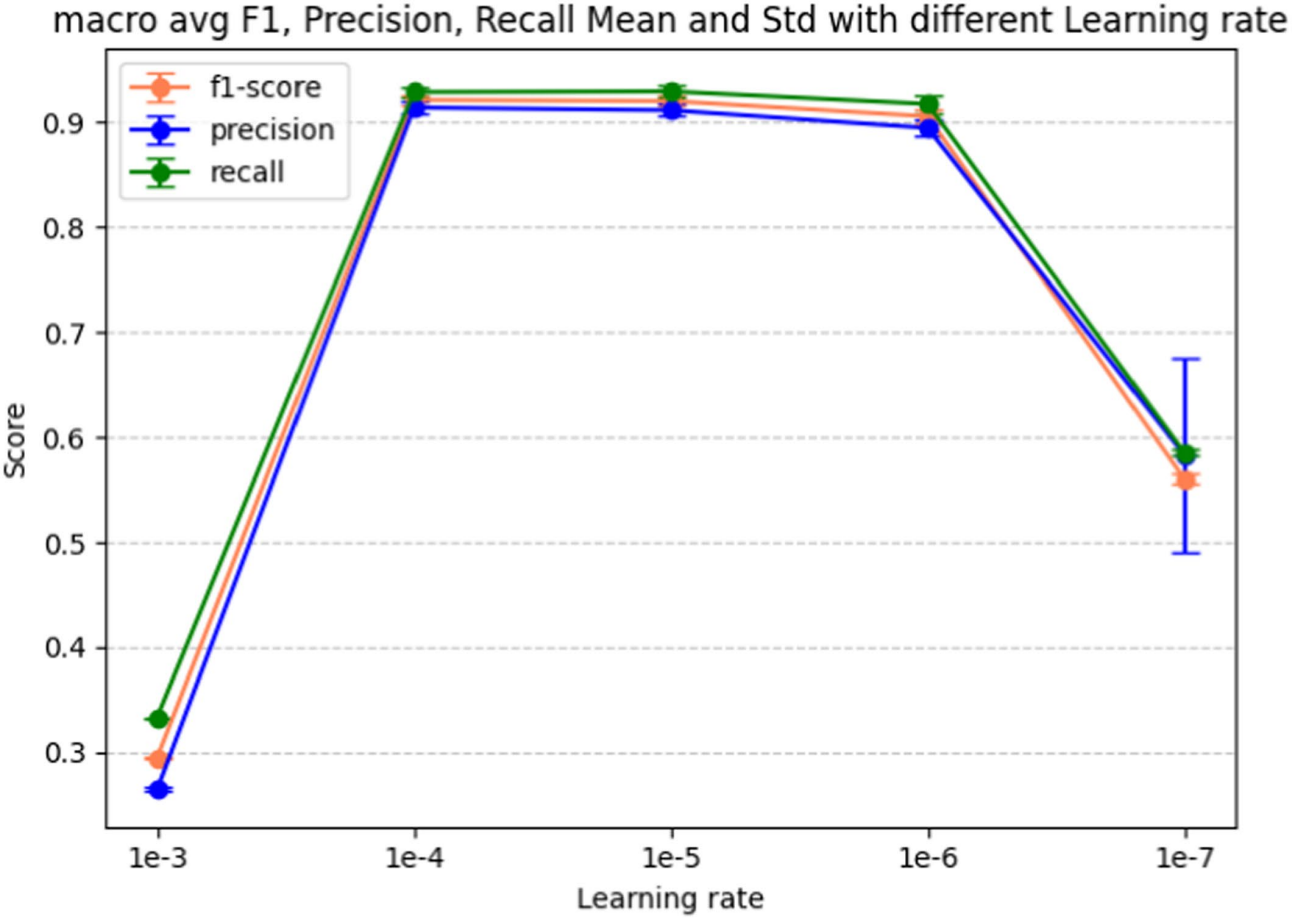
Macro F1, precision, recall score vs. learning rate for the BERT-based adverse event extraction model hyperparameter learning rate.

**Figure 10:**
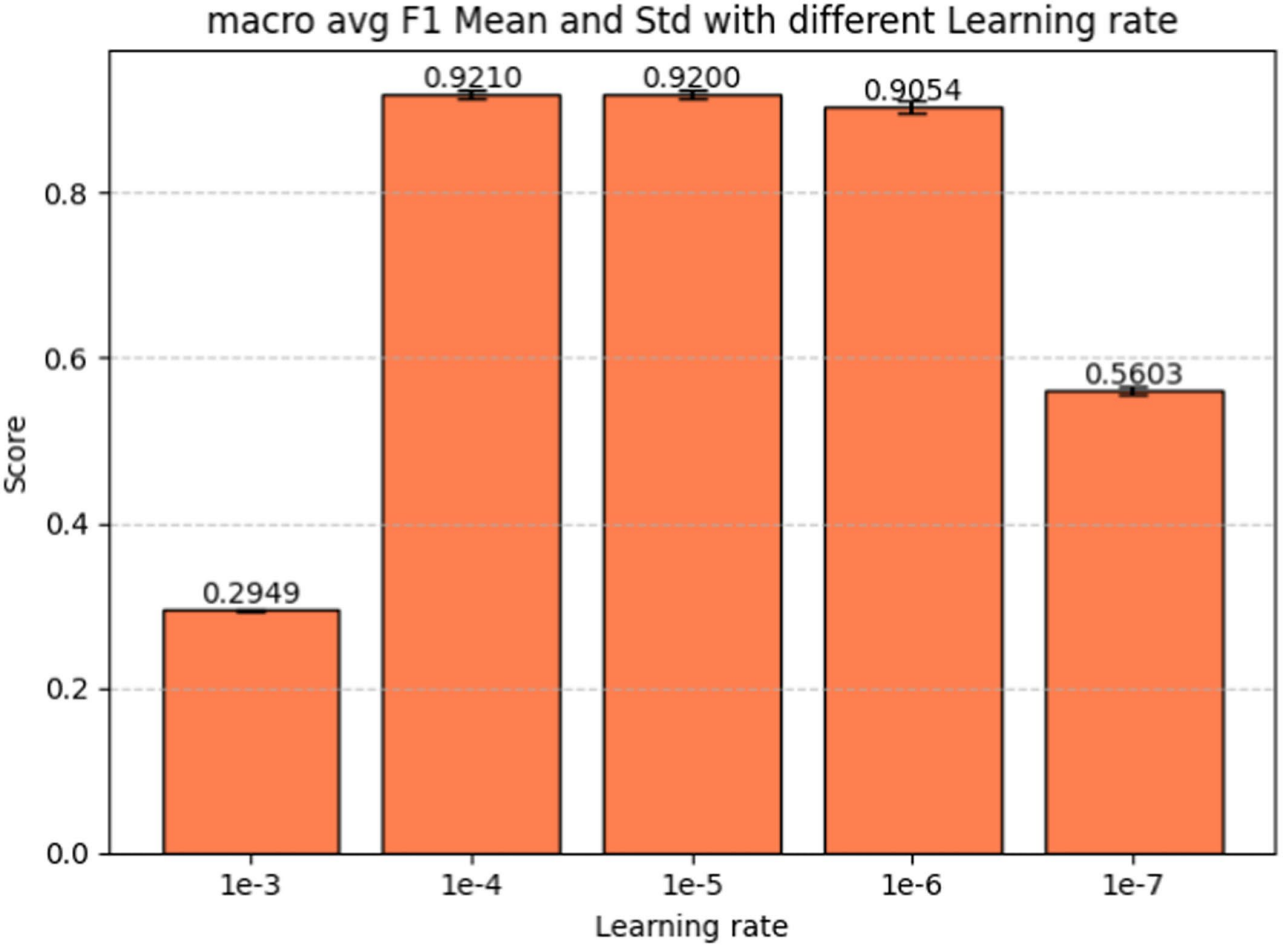
Macro average F1-score vs. learning rate for fine-tuning the BERT-based adverse event extraction model hyperparameter learning rate.

Learning rate is the last hyperparameter in fine-tuning our model. This parameter governs the extent to which the model weights adjust in response to the loss gradient. In Figure 9 we present the performance metrics (macro-average F1-score, precision, and recall) across various learning rates: 1e-3, 1e-4, 1e-5, 1e-6, and 1e-7. Error bars in the graph depict the standard deviation for each metric at the respective learning rate. Figure 10 shows the macro average F1-score across different learning rates in the BERT-based adverse event extraction model, with bars representing the mean F1-scores achieved for the five different learning rates.

A learning rate that is too large may lead the model to converge too rapidly, potentially setting on a suboptimal solution or even diverging. Conversely, a learning rate that is too small can extend the training process excessively, possibly failing to converge within a reasonable timeframe. After evaluating performance across different learning rates, a learning rate of 1e-5 emerged as the optimal choice. This learning rate is likely to yield the highest F1 score with minimal standard deviation, indicating stable and high-quality performance across different runs. Striking a balance between aggressiveness and conservatism, it avoids overshooting the minimum while ensuring an effective learning process. Consequently, this learning rate of 1e-5 was selected for model development, fostering effective learning and convergence to a robust solution that generalizes well to unseen data.

### 2.6 Evaluate metrics

For our BERT-based adverse event extraction model, we evaluated its performance using precision, recall, and the F1 score across three separate classes B-AE, I-AE, and O. Precision was calculated using Eq. 1 for each class. Here, *TP_i_* is the number of instances correctly predicted as class *i* (B-AE, I-AE, or O); *FP_i_* are the instances predicted as class i but actually not labeled as class *i*.


(1) $${\text{Precision}}_{i}=\frac{TP_{i}}{TP_{i}+FP_{i}}$$

Recall was calculated using Eq. 2 for each class. Here, *FN* are the instances labeled as class *i* but predicted as other class.


(2) $${\text{Recall}}_{i}=\frac{TP_{i}}{TP_{i}+FN_{i}}$$

F1 score is the weighted average of precision and recall and is calculated using Eq. 3. Therefore, *F*1 score takes both false positives and false negatives into account.


(3) $$F1_{i}=2*\frac{{\text{Precision}}_{i}*{\text{Recall}}_{i}}{{\text{Precision}}_{i}+{\text{Recall}}_{i}}$$

### 2.7 Model evaluation

#### 2.7.1 Internal evaluation on ADE-Corpus-V2 dataset

The 10-times hold-out evaluation is a robust internal validation method for assessing the performance of a BERT-based model for adverse event extraction on ADE-Corpus-V2 dataset. This method entails randomly splitting the dataset into 80% training and 20% testing sets ten times, with subsequent calculation of evaluation metrics for each split. The reported precision, recall, and F1 scores for the B-AE, I-AE, and O classes typically represent the average of these metrics over the 10 evaluations. This averaging accounts for variance in the model’s performance stemming from different dataset splits, providing a more robust estimate of the model’s true predictive capability.

This approach ensures that the results remain unaffected by particularly favorable or unfavorable dataset splits, providing a more generalized assessment of the model’s capability to accurately identify and classify each type of entity in the adverse event extraction task.

#### 2.7.2 External evaluation on SMM4H dataset

In the external evaluation phase of our study we utilized the entire ADE-Corpus-V2 dataset along with the best fine-tuned parameters to train a BERT-based model for adverse event extraction. Subsequent to the training, we externally evaluated the model on a dataset consisting of tweets from the SMM4H dataset. This evaluation employed a confusion matrix to visually represent the model’s predictions against the true labels. The matrix entries reflected the counts of true positives, false positives, and false negatives for the B-AE, I-AE, and O classes. Furthermore, precision, recall, and F1 scores were calculated to provide a quantitative measure of the model’s performance across these classes.

The model’s proficiency in extracting adverse events was further demonstrated through a table featuring examples of adverse event term extractions from tweets. This table illustrated various scenarios: “exactly recognized adverse event”, where the model accurately identified adverse events in accordance with human annotations; “miss recognized AE”, indicating instances where the model failed to detect an adverse event; “partially recognized adverse event”, cases where the model identified only a segment of an adverse event; and “recognized more than adverse event”, where the model identified extra terms beyond the actual adverse event. This qualitative analysis provided additional context to the quantitative metrics, enriching our comprehension of the model’s effectiveness in real-world adverse event identification from tweets.

## 3 Results

### 3.1 Internal evaluation result on ADE-Corpus-V2 data

**Figure 11:**
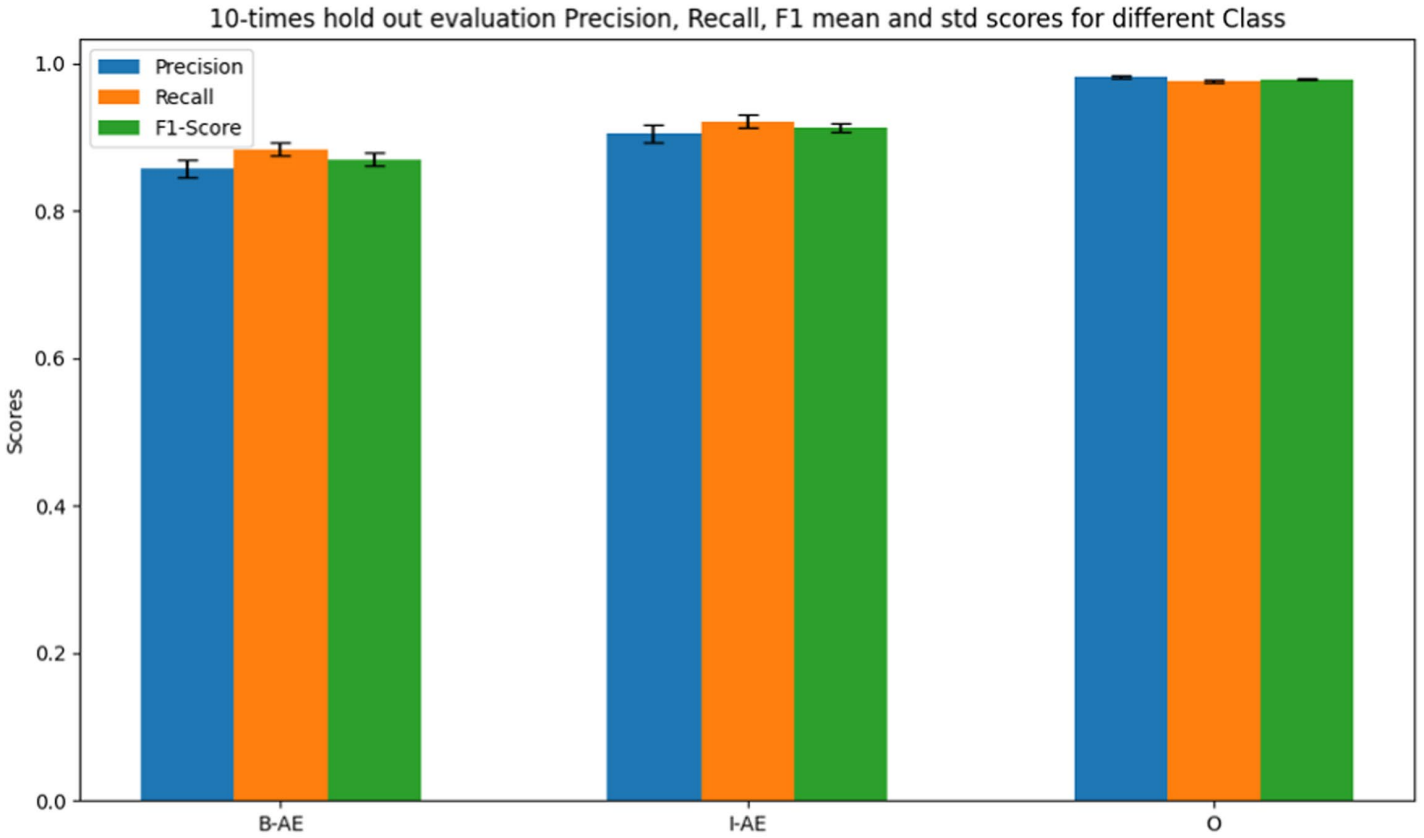
Performance metrics of the BERT-based adverse event extraction model from 10 times holdout internal evaluations on ADE_Corpus_V2 dataset.

Figure 11 shows the internal evaluation metrics through a10-times holdout for the BERT-based adverse event extraction model on ADE_Corpus_V2 dataset. The bars represent precision, recall, and F1-scores for the three classes: B-AE, I-AE, and O. Specifically, the model achieved an average F1 score of 0.8575 with a standard deviation of 0.0084 for the B-AE class, pertaining to words marking the beginning of an adverse event. For the I-AE class, indicative of words within an adverse event, the model recorded an average F1 score of 0.9049 with a standard deviation of 0.0066. Additionally, for the O class, representing words outside an adverse event context, the model demonstrated exceptional precision with an average F1 score of 0.9813 and a notably low standard deviation of 0.0013. The F1 scores signify the model’s robust and consistent performance across multiple evaluations during the internal testing phase.

### 3.2 External evaluation results on SMM4H dataset

**Figure 12:**
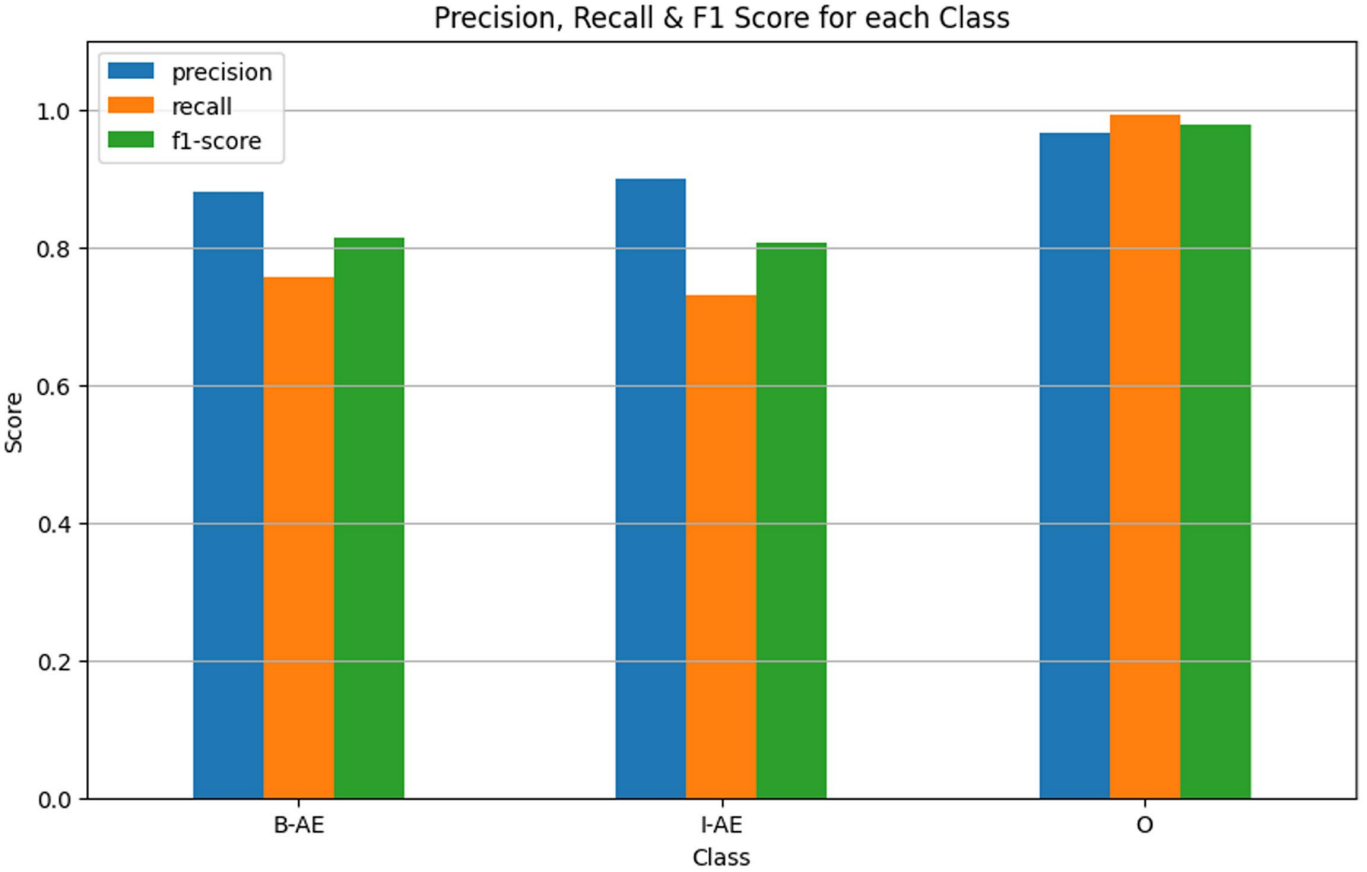
Performance metrics of the BERT-based adverse event extraction model from external evaluation on SMM4H dataset.

The external evaluation of the BERT-based model for adverse event extraction on the SMM4H dataset revealed promising results, as depicted in Figure 12. In terms of performance metrics, the model achieved F1 scores of 0.8127, 0.8068, and 0.9790 for the B-AE, I-AE, and O classes, respectively. These scores demonstrate the model’s effectiveness in accurately identifying adverse event-related information within tweets.

**Figure 13:**
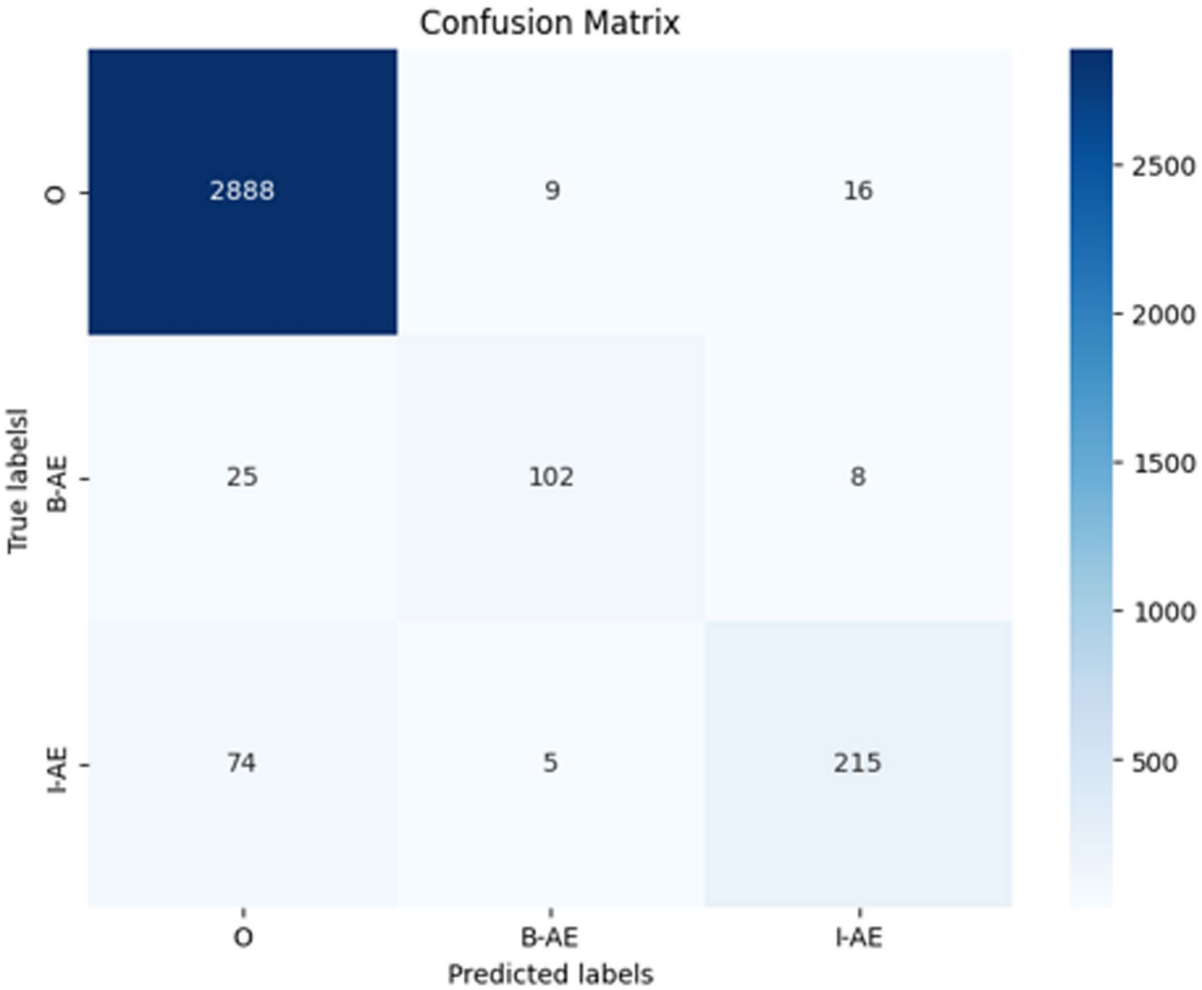
Confusion matrix of the external evaluation on SMM4H dataset for the BERT-based adverse event extraction model.

To facilitate a comprehensive understanding of these results, Figure 13 presents a confusion matrix illustrating the model’s classification accuracy across the predicted labels versus the true labels. The matrix showcases actual classes (true labels) in rows and predicted classes in columns. The main diagonal represents the number of correct predictions for each class, while off-diagonal elements reveal misclassifications, providing insight into the model’s specific strengths and weaknesses.

For the O class (tokens outside of an adverse event), the model correctly identified 2,888 instances (true positives). However, it incorrectly predicted 9 instances as B-AE and 16 as I-AE classes (false negatives for the O class). In the B-AE class (beginning of an adverse event), there were 102 true positives, indicating correct identification. However, the model misclassified 25 instances as O class and 8 as I-AE class (false negatives for B-AE class). For the I-AE class, the model correctly identified 215 instances but misclassified 74 instances as O class and 5 as B-AE class (false negatives for I-AE class).

In addition, Table 2 provides a detailed comparative analysis of the BERT-based model’s performance in extracting adverse event terms from tweet data. The table contrasts the ground truth (human-labeled, indicated in bold and italic) with the model’s predictions (underlined). In the exactly recognized adverse event case, the model’s prediction aligns precisely with human annotation, as seen in the example where both identify the adverse event of “frontal headache”. The miss recognized adverse event category highlights instances where the model failed to detect an adverse event, such as “sick”. In the partially recognized adverse event scenario, the model correctly identified a portion of the adverse event terms, exemplified by recognizing “rapid cycling”, which is part of the human labeled adverse event “triggers my rapid cycling”. Lastly, the recognized more than adverse event case shows instances where the model predicted additional adverse events not labeled by human, illustrated by identifying adverse event of “going crazy” that are not labeled by human.

**Table 2:** Cases of comparing model-extracted adverse event (underlined) with human-labeled adverse event (bold and italic).

Case	Example in tweets
Exactly recognized adverse event	06.30 day 14 Rivaroxaban diary. Thanks to paracetamol and hot water bottle I had 4 h continuous sleep. Woke up with **frontal headache**, 1 2
Miss recognized adverse event	rt my philly dr prescribed me trazodone,1pill made me so fkn**sick**, couldnt move 2 day.xtreme**migraine*****, puke, shakes***. any1else
Partially recognized adverse event	well I am taking it with a mood stabilizer (lamictal). i cannot take anti-depressants by themselves-**triggers my rapid cycling**
Recognized more than adverse event	does cipro make anyone’s else’s **brain turn to mush** or am i actually just going crazy?

## 4 Discussion

Machine learning techniques have found widespread application in numerous fields for processing structured data (21–34). However, when dealing with unstructured data, a unique set of methodologies is required due to the inherent nature of this data type (35–39). Unlike structured data, unstructured data lacks a predefined model and encompasses types such as text, images, audio, and video, which do not neatly fit into conventional databases. Extracting features from unstructured data is a more intricate process, often employing techniques like NLP for text analysis. Notably, data quality emerges as a critical concern, particularly evident in scientific fields like genomics (40–42). Unstructured data tends to have lower quality, necessitating cleaning procedures such as noise reduction and rectification of transcription errors in text.

Machine learning and deep learning applied to unstructured data demand distinct methodologies, algorithms, and preprocessing techniques owing to the unique characteristics of this data type. The chosen approach is contingent upon the specific nature of the data and the objectives of the machine learning and deep learning tasks at hand. For textual data, BERT stands out as a revolutionary NLP model. Its proficiency in capturing contextual information allows it to comprehend the intricacies of language exceptionally well, leading to superior performance across various NLP tasks. There are several variants of the BERT model, each tailored for specific purposes or devised to address particular limitations of the original model (43–47). The selection of the appropriate BERT-based model depends on various factors, including the nature of the task, dataset size, computational resources, and language requirements.

In our study we opted for the BERT-based-uncased model with 12 transformers layers and approximately 110 million parameters. This decision was motivated by the model’s optimal balance between size and performance—its architecture is adequately comprehensive to capture contextual nuances without the computational intensity associated with larger models like BERT-large which includes 24 transformer layers and about 340 million parameters. The uncased variant treats the text as lowercase, a beneficial characteristic for social media text that often lacks consistent capitalization.

While BERT-large offers increased power, it demands substantial memory and extended training times, posing practical challenges in certain research environments. Domain-specific BERT like BioBERT or SciBERT, pre-trained on specialized corpora, may outperform the base model in relevant tasks but could exhibit overfitting or underperform on more general datasets.

Given that our model was trained on the ADE-Corpus-V2 dataset in the medical reports domain and externally tested with social media tweets data, a more general BERT model like BERT-based-uncased is recommended for our study.

Utilizing a high-performance computing (HPC) environment with an NVIDIA V100 GPU and 32GB of memory, we efficiently fine-tuned our BERT-based model for adverse event extraction in a mere 4 h. This process involved a delicate balance between hyperparameters and training time. While augmenting epochs generally improves model accuracy, our focus was on preventing overfitting by stopping training once the model reached convergence.

The selected batch size was sufficiently large to maximize the processing power of V100 GPU without surpassing its memory limits. In addition, the learning rate was carefully set to ensure quick yet stable convergence. This thoughtful approach to selecting epochs, batch size, and learning rate played a pivotal role in optimizing our training procedure for both speed and performance, leveraging the capabilities of the HPC environment and the NVIDIA V100 GPU to their fullest potential.

The advent of models like BERT has markedly improved our capability to comprehend and extract context from text. However, a significant challenge persists in creating models that generalize effectively across diverse social media platforms and data types. The real-time processing and extraction of adverse events from social media remain computationally intensive and not fully realized. Additionally, the manual annotation process for adverse events in social media data is time-consuming and demands domain expertise to generate high-quality human-labeled data.

The presence of informal language, including slang, misspellings, and creative language use in social media content, poses a formidable obstacle for models to accurately interpret the data. In summary, despite the strides made in developing BERT-based models for adverse event extraction from social media, these enduring challenges remain critical in the field of pharmacovigilance research.

Social media data has been acknowledged to exhibit demographic or geographic biases, which can impact the model’s generalizability (48). It’s important to note the potential bias inherent in both the human annotated ADE-corpus-V2 (training dataset) and the SMM4H social media tweets (evaluation dataset). This limitation may influence the model’s ability to generalize its performance to broader populations or across different social media platforms. Looking ahead, as larger human annotated adverse event datasets become available, encompassing a wider spectrum of populations, there is an opportunity to retrain and evaluate our model. By doing so, we can work towards mitigating the risk of bias and improving the generalizability of model performance.

The quality of annotated adverse event data is pivotal for the effectiveness of our model. Annotating social media data presents distinct challenges compared to traditional medical report data due to the inherent ambiguity in user-generated tweets, which often include slang or colloquial language. The SMM4H annotated adverse event dataset, utilized in our study, was meticulously curated by a team of trained medical experts. These experts systematically labeled tweets related to adverse drug events following established guidelines, ensuring both consistency and accuracy in annotations. Moreover, their expertise allowed them to adeptly navigate the nuances of informal language and slang, thereby ensuring that annotations faithfully captured the medical context despite the informal nature of social media posts (49). To address the unique challenges associated with social media annotation, we advocate for collaborative efforts among researchers, clinicians, and linguists. Establishing standardized guidelines for annotating social media data through such collaborative endeavors will be pivotal in ensuring the quality and reliability of annotations moving forward.

Numerous studies have highlighted the lack of interpretability in deep learning models, including BERT, raising concerns about the transparency and trustworthiness of these models (50). Attention mechanisms represent a promising avenue for shedding light on the inner workings of the model by elucidating significant features within the input text. By visualizing the attention weights, we aim to gain a deeper understanding of how the model processes and prioritizes information. Furthermore, visualization techniques such as Grad-CAM (51) and SHAP (52) offer additional insight by identifying the regions of the input data that are most influential for BERT’s predictions.

Optimizing hyperparameters is indeed crucial for achieving optimal performance. However, we recognize that training and fine-tuning BERT models demand significant computational resources and time, posing a challenge for deploying them in real-world pharmacovigilance settings with resource constraints. To tackle these scalability challenges, several strategies could be employed. First, adopting efficient training techniques can help streamline the training process and reduce computational requirements. Additionally, model compression techniques, such as lighter models like DistilBERT, can mitigate the computational burden while maintaining performance. Furthermore, utilizing cloud computing resources offers scalability and flexibility, enabling the deployment of BERT-based models in resource-constrained environments. By implementing these strategies, the scalability and practicality of deploying BERT-based models could be enhanced for pharmacovigilance in real-world settings.

It is crucial to address privacy concerns and ethical considerations when utilizing social media data for pharmacovigilance. Our study exclusively involved the extraction of text information from tweets, without accessing any personal user details. This approach allowed us to harness the valuable data available on social media for pharmacovigilance purposes while safeguarding user privacy.

Considering the long-term performance and adaptability of our BERT-based model to evolving social media dynamics is paramount. To address this aspect, we plan to implement long-term learning strategies such as continuous training on updates of the training data to ensure the model’s sustained performance over time. Additionally, collaborating with domain experts in pharmacovigilance to review our model’s predictions and provide feedback will be instrumental in enhancing its adaptability to new adverse events and emerging trends. These measures will help to ensure the robustness and relevance of our model in real-world applications beyond immediate performance evaluations.

Addressing the challenges of applying research findings to real-world situations is paramount. Utilizing BERT-based models in pharmacovigilance for drug safety requires careful planning and consideration of various factors. This includes integration with existing systems, adherence to regulatory guidelines such as those from the FDA, and ensuring user acceptance. It is essential for the model to seamlessly integrate with current pharmacovigilance systems while meeting data privacy, validation, and transparency standards. Additionally, garnering acceptance from social media users is crucial for the model’s effectiveness. Moving forward, our efforts will focus on devising strategies to overcome these challenges and ensure the model’s utility in clinical and regulatory settings. By addressing practical considerations and potential barriers upfront, we aim to facilitate the seamless integration and adoption of our BERT-based model in real-world applications.

## 5 Conclusion

In conclusion, this research underscores the significance of social media platforms as valuable resources for monitoring health-related information and adverse events associated with medications and treatments in drug safety surveillance. Despite their potential, accurately and efficiently extracting drug adverse events from social media remains a challenge in both NLP research and the pharmacovigilance domain. Recognizing a gap in the detailed implementation and evaluation of BERT-based models for drug adverse event extraction on social media, our study addresses this issue by developing a specialized BERT-based language model. Leveraging publicly available labeled adverse event data from the ADE-Corpus-V2, we optimized key hyperparameters during model construction. Through ten hold-out evaluations on ADE-Corpus-V2 data and external validation using human-labeled adverse event tweets data from SMM4H, our model consistently demonstrated high accuracy in drug-related adverse event detection. This study not only highlights the efficacy of BERT-based language models in identifying drug-related adverse events in the dynamic landscape of social media data, but also emphasizes the importance of a comprehensive implementation study design and evaluation. In doing so, our research contributes to advancing pharmacovigilance practices and methodologies, particularly in the context of emerging information sources like social media.

## Data availability statement

Publicly available datasets were analyzed in this study. This data can be found here: https://huggingface.co/datasets/ade_corpus_v2.

## Author contributions

FD: Writing – original draft, Writing – review & editing, Conceptualization, Data curation, Formal analysis, Methodology, Software, Validation, Visualization. WG: Data curation, Formal analysis, Writing – review & editing. JL: Writing – review & editing. TP: Writing – review & editing. HH: Conceptualization, Methodology, Project administration, Supervision, Writing – review & editing.
